# A Fuzzy-Based System for Autonomous Unmanned Aerial Vehicle Ship Deck Landing

**DOI:** 10.3390/s24020680

**Published:** 2024-01-21

**Authors:** Ioannis Tsitses, Paraskevi Zacharia, Elias Xidias, Michail Papoutsidakis

**Affiliations:** 1 Department of Industrial Design and Production Engineering, University of West Attica, 12241 Egaleo, Greece; mscdrones8096626@uniwa.gr (I.T.); mipapou@uniwa.gr (M.P.); 2 Department of Product & Systems Design Engineering, University of the Aegean, 84100 Syros, Greece; xidias@aegean.gr

**Keywords:** unmanned aerial vehicles, autonomous ship deck landing, fuzzy logic control system

## Abstract

This paper introduces a fuzzy logic-based autonomous ship deck landing system for fixed-wing unmanned aerial vehicles (UAVs). The ship is assumed to maintain a constant course and speed. The aim of this fuzzy logic landing model is to simplify the task of landing UAVs on moving ships in challenging maritime conditions, relieving operators from this demanding task. The designed UAV ship deck landing model is based on a fuzzy logic system (FLS), which comprises three interconnected subsystems (speed, lateral motion, and altitude components). Each subsystem consists of three inputs and one output incorporating various fuzzy rules to account for external factors during ship deck landings. Specifically, the FLS receives five inputs: the range from the deck, the relative wind direction and speed, the airspeed, and the UAV’s flight altitude. The FLS outputs provide data on the speed of the UAV relative to the ship’s velocity, the bank angle (BA), and the angle of descent (AOD) of the UAV. The performance of the designed intelligent ship deck landing system was evaluated using the standard configuration of MATLAB Fuzzy Toolbox.

## 1. Introduction

Today, the utilization of drones in the maritime domain is rapidly increasing. Their uses for civil missions, as well as military operations, vary greatly. Regarding the civil sector, drones—especially UAVs—are used for transporting spare parts, documents, medicine, etc., between land and ships at sea or between ships at sea only [1], controlling traffic and the emission of pollutants from ships, and even preventing illegal activities such as piracy, smuggling, and illegal fishing [2]. The underlying reason for this fact lies in the drone’s numerous sensing capabilities and interchangeable payload equipment [3] combined with the reduced risk, time, and cost that the UAVs provide in a wide range of missions [1].

On the other hand, in the military/security domain, another vast range of missions is covered solely by UAVs, both in peacetime and in wartime periods. Drones provide unparalleled surveillance capabilities for border security, facilitating real-time monitoring of large areas with minimal risk to individuals on the ground, and they have the ability to deploy easily and respond immediately to any potential threat or emergency [2]. Furthermore, drones are used for search and rescue (SAR) missions, improving response times and limiting exposure to dangerous conditions. They can even be used to drop flotation devices for lifeguarding purposes [3]. Additionally, UAVs are mostly used in maritime surveillance operations to uphold the safety and security of maritime traffic, while concurrently thwarting illicit activities. UAVs are also used as moving targets during military exercises of naval forces.

Lastly, in wartime, the operation capabilities of modern maritime military UAVs experience further augmentation. Depending on the payload and the equipment of the UAV, drones are able to undertake strategic missions. Recognizing the value of utilizing UAVs in the maritime domain, the U.S. Navy, as well as the U.K. Royal Navy [1], have integrated a variety of different drones in their arsenal, leading the way in modern warfare and naval research technology.

Landing, one of the most critical parts of the flight of a drone, is a topic that has been scarcely researched by the academic community. Even today, most UAV landings are performed manually by their operators from inside the drone’s ground station, depriving the drone pilot of peripheral vision, instinctive senses, and space perception during the procedure. As a result, the majority of UAV’s crashes happen due to human errors during takeoffs and landings, which constitute the most dangerous parts of a drone’s flight [4].

More specifically, during ship deck landings, the difficulty of manually guiding the UAV to touchdown increases exponentially, demanding even more experience and skill from the drone pilot. The unpredictable nature of the maritime environment, which is characterized by low visibility, strong winds, high humidity and noticeable waves, in combination with the dynamic motion of the landing platform on the ship in the sea with its limited dimensions, pose a great, sometimes even impossible, challenge for the drone pilot. Consequently, the imperative arises for the development of an autonomous landing system, which has the potential to crucially contribute to the progression of drone technology within the maritime sector.

Concerning the operation of UAVs, relevant studies exist that have explored autonomous flight navigation; nonetheless, a very limited number of sources have focused on autonomous UAV landings. In [5], a review of vision-based autonomous landing systems for UAVs is introduced, classifying the existing techniques into static, dynamic, and complex scenes. The work of [6] presents an extensive review of various landing techniques using a number of control- and vision-based techniques for different types of aircraft. In [7], the control of the landing and descent simulation of a Boeing 747, employing its linearized landing configuration model, is governed using fuzzy logic controllers (FLCs). The rule bases defining the FLCs are formulated as functions of the linearized model’s inputs, specifically the vertical velocity and altitude of the Boeing 747. The outputs are the elevator and throttle deflections. The work of [8] proposes an autonomous system of a UAV docking station consisting of an electromagnet docking structure, auto-landing, and visual servo control.

In [4], a control system for autonomous flight and landing based on fuzzy logic is introduced. The system consists of three fuzzy logic controllers for autonomous navigation in UAVs, and three additional fuzzy logic modules are created within the primary landing system. These modules control the horizontal and vertical positions of the aircraft relative to the runway under a tactical air navigation approach. The work in [9] introduces a technique for auto-landing using fuzzy logic-based position and speed control. This system utilizes position and velocity data to regulate the altitude of a quadcopter UAV. The velocity control algorithm is designed to facilitate a secure landing, preventing the quadcopter from impacting the ground at high speeds. Simultaneously, the position control component assesses altitude, ensuring a smooth and rapid landing. The integration of position and speed control enhances efficiency by minimizing landing time and offers increased safety assurances compared with conventional controllers.

The authors in [10] present a proposed fuzzy logic framework for intelligent aircraft landing decision systems. Various factors influencing the required aircraft landing decisions are incorporated in this approach, encompassing wind velocity, wind direction, visibility, and pilot experience. The landing decision process is categorized into three classes: feasible, cautious, and not feasible. The work of [11] proposes a hierarchical sliding mode-based adaptive fuzzy control for underactuated nonlinear systems, such as a UAV, in which there is a mismatch between the control input and the degrees of freedom being controlled that increases the difficulty of control. In [12], a decision support system is introduced utilizing fuzzy logic to aid the pilots of Boeing 747-100 aircraft. The research aims to mitigate accidents associated with the aircraft landing assistance system and conduct a speed analysis of the Boeing 747-100. To prevent aviation accidents, factors such as aircraft landing speed, braking force, rolling resistance, distance to obstacles, and runway length are meticulously considered.

This paper focuses on the concluding stages of UAV operations, specifically the final approach and touchdown periods. It introduces a fuzzy logic-based autonomous ship deck landing system for fixed-wing UAVs as a proposed solution to most of the previously mentioned complex problems. The proposed strategy is based on the development of an intelligent ship deck landing system based on a fuzzy logic approach. The novelty of the work in comparison with the literature mentioned above is summarized around five major points:

1. The analysis presented in this paper focuses on the landing of UAVs in the maritime domain. The challenging conditions and dynamic nature of the sea demand greater precision and maneuverability for the drone, setting a more demanding context than a standard ground landing, as presented in the majority of the bibliography.
2. The utilization of RF sensors (TDOA localization mechanism and RFID system) instead of other vision-based systems or image processing algorithms like those proposed in [4,6,8] to counter the negative impact of water mist and sea surface phenomena on the image quality of the visual sensors.
3. Two new fuzzy inputs are presented, highlighting the importance of calculating as many factors as possible in the autonomous ship deck landing system. First, the range of the UAV from the landing deck (RLD) is measured using different localization systems and used as an input, both in the altitude and the speed components of the FLS. Second, the concept of the indicated airspeed (IAS) is introduced (Section 2.1), as it affects immediately the aerodynamic profile of the UAV and, consequently, the ability of the drone to make vertical or horizontal turns.
4. The electronic warfare (EW)-resistant mechanism of the UAV’s landing system (Section 3.5) is based upon the deliberate omission of a GPS localization system and the utilization of short-range UHF sensors, pinpointing the exact location of the approaching drone.
5. This paper extends beyond the scope of the FLS, presenting a realistic, feasible, and integrated solution for UAV ship landing systems by incorporating sophisticated localization systems such as TDOA and RFID mechanisms, as well as a quick-recovery landing subsystem.

The paper is organized as follows: Section 2 presents the fundamental concepts for autonomous ship deck landing. Section 3 provides a concise overview of the main characteristics of the landing, as well as the sensors used for a successful landing process. Section 4 analyzes the developed fuzzy ship deck landing system, which comprises three fuzzy subsystems, and the simulation results are presented in Section 5. Finally, conclusions and directions for further research are presented in Section 6.

## 2. Fundamental Terms for Autonomous Ship Deck Landing

This section introduces fundamental terms related to the autonomous ship deck landing system, providing a comprehensive clarification of essential concepts and information.

### 2.1. Indicated Airspeed

IAS is the speed of the UAV relative to the body of air through which it is flying [13]. It is measured using an airspeed sensor and expressed in knots. In other terms, the IAS is “the speed of an aircraft as shown on its pitot static airspeed indicator, calibrated to reflect standard atmosphere adiabatic compressible flow at sea level, uncorrected for airspeed system errors” [14]. Basically, IAS represents the dynamic pressure on the airspeed sensor as the aircraft moves through a body of air. It is a function of the dynamic pressure experienced by the UAV and the atmospheric density of air surrounding the drone at a certain altitude. In practice, however, air density is considered independent of altitude due to the very low heights during landing. As a result, air density is considered to be constant, so there is no need for a dynamic pressure sensor to compute airspeed [15]. IAS is calculated using an airspeed sensor.

IAS is the most important speed of the pilot from an aerodynamic point of view, especially when controlling the aircraft during takeoffs or landings. It differs completely from the ground speed, which is the actual aircraft speed in relation to the ground. For fixed-wing aircrafts, such as the UAVs, it is the IAS that guarantees lift—not ground speed. So, for example, if airspeed is minimum, then stability conditions are severe and maneuvering abilities are limited [15].

### 2.2. Azimuth Angle of Wind and Relative Wind Speed

The azimuth angle of wind (AAW) is the direction from which the wind is blowing, measured in degrees clockwise from north on an azimuth circle. An azimuth circle consists of 360 degrees [16]. Traditionally, wind direction is reported as one of eight compass points (N, NE, E, SE, S, SW, W, NW) [16], which depicts certain degrees in the azimuth circle.

**Figure 1:**
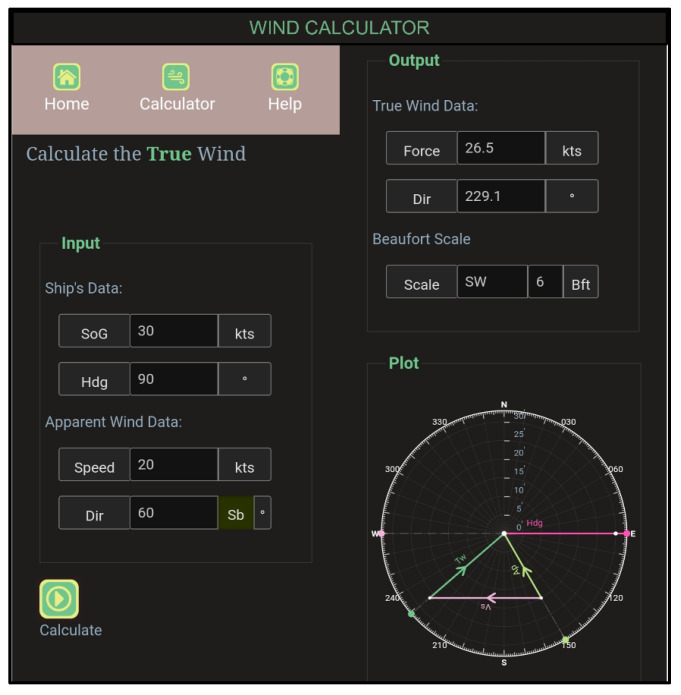
Wind calculator app.

The AAW is either true or relative, depending on the north used as a reference. True north corresponds with the direction indicated by a gyroscopic compass and is represented on a map as the line of longitude that converges on the North Pole [17]. Conversely, relative wind is calculated using relative north as a reference (Figure 1). In the case of a fixed-wing UAV, relative north is the nose of the UAV. Consequently, relative AAW signifies the wind direction relative to the UAV. In the FLS later, we utilize only relative AAW.

The speed of the wind (WS) [18] is also characterized as true WS and relative WS. True WS (ground wind) “is the actual speed of the wind as it passes over land or the surface of the sea” [19]. Relative or apparent wind speed (RWS) is the wind that a body (aircraft, UAV, ship etc.) “feels” as it moves through space. The AAW, as well as the WS, are measured using an anemometer providing their relative values.

### 2.3. Range from Landing Deck

**Figure 2:**
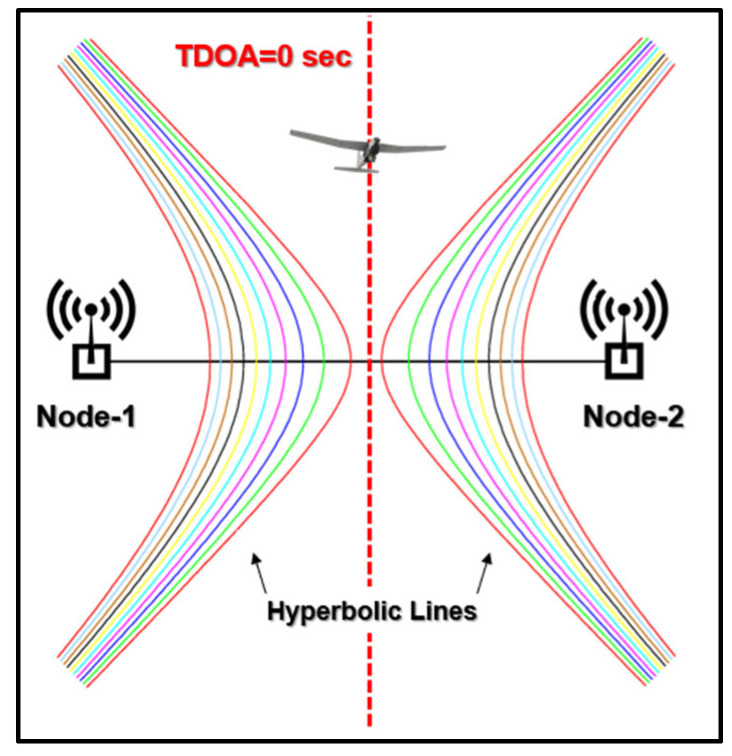
A TDOA-based localization mechanism with two nodes.

The RLD is measured in meters using a time difference of arrival (TDOA)-based localization mechanism. The working principle of the TDOA system is as follows: A modulated signal is transmitted from the UAV, and this signal is captured at three or more probes placed in different locations around the landing deck. Then, the signal captured at each receiver is shifted in time to locate a position of maximum correlation, and this time delay is later multiplied by the speed of light, calculating the distance difference between each probe. Afterward, the distance difference is plotted as a set of hyperbolic lines between the pairs of the probes (Figure 2), and lastly, the intersection of the lines indicates the location of the emitting drone [20]. To locate the UAV in the 2D plane, a three-node TDOA system is required. In this study, a four-node TDOA localization system is utilized to pinpoint the location of the UAV in the 3D space as the UAV approaches the ship.

**Figure 3:**
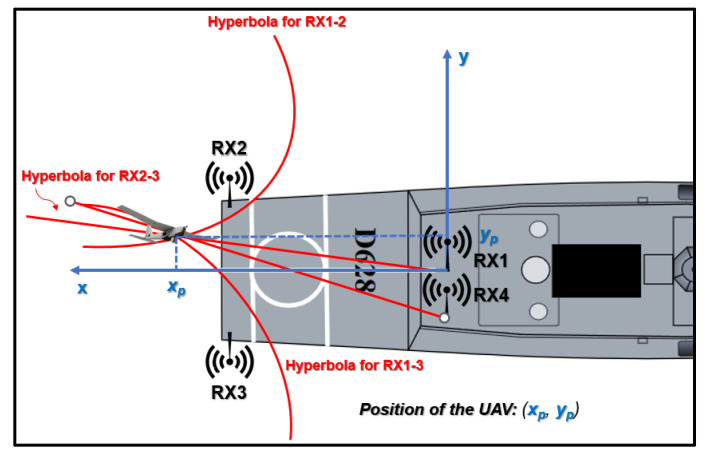
TDOA-based three-node system to calculate the distance of the UAV in the 2D space.

**Figure 4:**
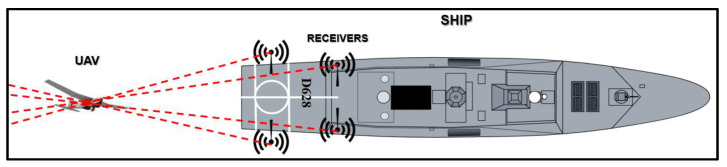
The TDOA-based localization four-node system.

To determine the drone’s distance from the ship, precise knowledge of the UAV’s location in the 2D plane is essential (Figure 3). This localization necessitates the deployment of a three-node TDOA-based system. In the context of this paper, however, a four-node TDOA system is employed to pinpoint the UAV within three-dimensional space (Figure 4). This configuration allows for the determination of both the RLD and altitude of the UAV, as detailed in subsequent sections.

Furthermore, because the times of arrival of the signal are measured using different receivers in the TDOA-based localization mechanism, precise synchronization between these nodes is essential [21]. Also, TDOA is vulnerable to time delay because the system’s measurements are based on the assumption that the signal travels the shortest path from the source (the emitting UAV) to the receiver (node) [22].

### 2.4. Altitude

The altitude of the UAV is the distance from the surface of the sea. It is measured in feet (ft) using a barometric altimeter, which measures atmospheric pressure to estimate altitude, combined with the 3D TDOA-based localization system. However, weather conditions can affect atmospheric pressure, which is why altimeters are often calibrated at the takeoff site. Also, to increase the accuracy of the height value, a four-node TDOA-based mechanism is utilized [23] to providing the position of the UAV in 3D space during the entire landing phase.

### 2.5. Relative Speed of the Drone

The relative speed of the drone (RSD) is the speed of the UAV in reference to the speed of the ship. More specifically, during the landing phase, it represents the difference between the SOG of the UAV and the SOG of the ship as the UAV approaches the landing deck of the ship, measured in knots. In the context of this paper, where the ship moves in front of the drone, it is calculated by subtracting the constant SOG of the ship from the SOG of the UAV.

### 2.6. Bank Angle

The BA is the angle of turn in the azimuth plane of an aircraft and is measured in degrees (°) [24]. In other terms, it is the angle between the aircraft’s normal, or vertical, axis and the earth’s vertical plane containing the aircraft’s longitudinal axis. The BA of an aircraft is measured from 0° to 179° port or starboard (left or right) [25]. When an aircraft makes a turn, it banks to one side, and the BA is the amount by which the aircraft is tilted. It is dependent on the forces of lift and weight that are acted upon the UAV.

### 2.7. Angle of Descent

The AOD is the angle between the aircraft’s horizontal axis and the earth’s horizontal plane. It indicates how steep the “dive” of the aircraft is during the landing or takeoff phase, and it is measured in degrees.

## 3. The Autonomous Landing Strategy

### 3.1. Ship Characteristics and Sensors

**Figure 5:**
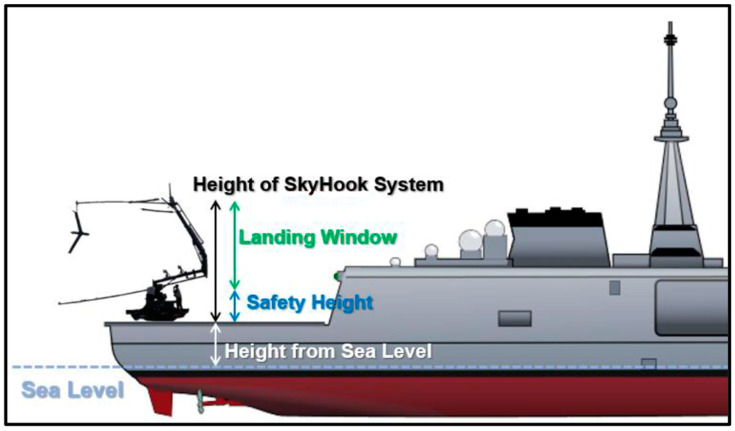
Different heights used for the landing system.

The ship’s course over ground (COG) and SOG are considered to be constant during the landing phase of the drone. Due to the limited dimensions of the helidecks on most ships, the most successful landing of a fixed-wing UAV would involve the use of a vertical rope or a net to “catch” the flying drone. In this context, “SkyHook” [26] is proposed as a capture system onboard the helideck of the ship. Also, we consider the height level of the ship’s landing deck from sea surface to be 8 m (Figure 5).

Additionally, four spatially separated nodes of the TDOA-based localization mechanism are planted in different positions on the landing deck to track the UAV’s position in the three-dimensional space during its approach to the ship [27]. The ultimate goal of the autonomous landing system is to present an alternative navigation approach that operates independently of a global positioning system (GPS), providing a robust and self-reliant solution in a contested electromagnetic environment.

**Figure 6:**
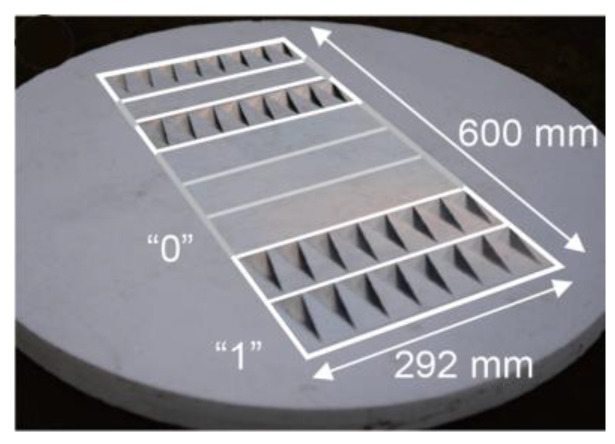
The chipless RFID tag used in the MilliSign System [28].

**Figure 7:**
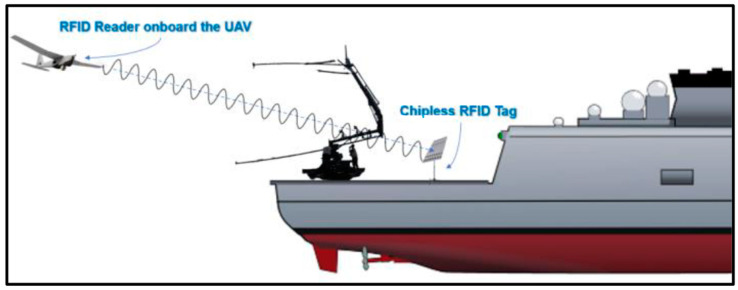
The chipless RFID tag on a tripod stand behind the SkyHook landing system.

For even better accuracy and precision during the last phase of the landing procedure, in a RLD less than 20 m, the use of a passive ultra-high frequency (UHF) radio frequency identification (RFID)-based UAV positioning system is proposed [21]. The best candidate RFID for this role is the MilliSign guidance system based on a batteryless tag to support UAVs in poor visibility and all-weather conditions [28]. A corner reflector (CR) array-based chipless RFID tag and a one-shot slant range reading procedure with commercial off-the-shelf (COTS) mmWave radar constitute the MilliSign system, as shown in Figure 6. The RFID tag is placed vertically in the longitudinal axis of the landing deck, right behind the receiver rope of the “Skyhook” capture system. This placement is designed to facilitate the alignment of the fixed-wing UAV with the “catch rope” in the final meters of its flight. (Figure 7). The chipless tag is a conventional high radar cross-section (RCS) scatterer with retro-reflective attributes for 3D incident wave and provides a wide 3D read range so that it can be read by the UAV’s UHF radar from a distance of more than 10 m, with a viewing angle of more than 30 in elevation and azimuth. The tag is covered by a radome, as it is sensitive to debris, such as dust, sand, mud, and water (rain), which can enter and deteriorate the backscattering RCS of the tag. The RFID reader (mmWave radar), on the other hand, is deployed on the UAV, and the communication between the reader and the tag is much more efficient in LOS.

Lastly, as a general observation, the majority of merchant ships often cruise at 18 to 25 knots. However, warships are capable of operating at speeds of approximately 32 knots. In this particular context, the designated maximum speed for the ship during the landing phase was stipulated as 30 knots. Subsequently, this value serves as the basis for determining the maximum RSD.

### 3.2. UAV Characteristics and Sensors

**Figure 8:**
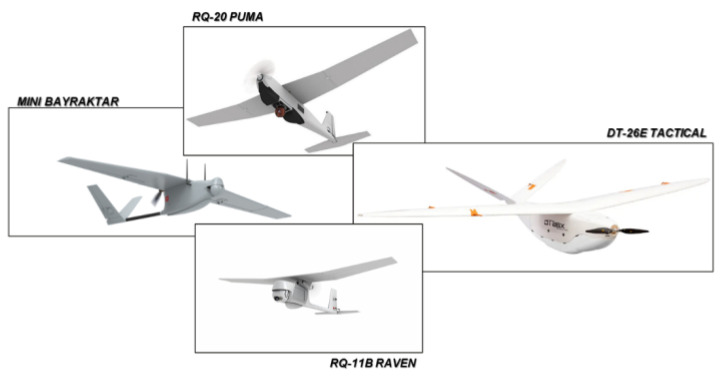
Examples of small- to medium-sized/class UAVs.

The fixed-wing UAV that is investigated in this paper is a small- to medium-sized/class drone (Figure 8) capable of reaching a maximum speed of 70 knots during the landing phase of its mission. Also, the BA of the UAV is considered to be a maximum of 60 degrees, which is indicative of a highly agile military UAV capable of executing sharp and steep turns. However, the ability of the UAV to turn at such degrees is immediately dependent on the IAS, which strongly affects the aerodynamics of the UAV.

Additionally, to enable precise landing, the UAV necessitates an array of sensors and equipment. The sensors of the fixed-wing UAV gather the information from the environment of the drone and send it to the microcontroller of the UAV. For the purpose of the autonomous ship deck landing proposed in this paper, the UAV should be equipped with the following sensors:

- An anemometer
- An airspeed sensor
- A barometric altimeter
- An inertial navigation system (INS)
- A radio frequency (RF) transmitter for the TDOA-based localization system
- A mmWave radar (RFID reader)

Regarding the RFID reader used on the UAV, its small size, lightweight design, and cost-effectiveness, as well as its minimal power requirements contributed to enhancing the UAV’s autonomous capabilities [28].

All these sensors constitute the essential equipment of a fixed-UAV to conduct a successful autonomous ship deck landing as described in this work. The deliberate omission of a GPS sensor during the landing phase is driven by the necessity for the UAV to exhibit resistance against electronic warfare interference (see Section 3.5).

### 3.3. The Main Attributes of Landing

The scope of this paper is the design of a precise autonomous landing system utilizing fuzzy logic to be used by fixed-wing UAVs during ship deck landings. For a successful landing to take place, three variables must be taken into consideration [4]. First of all, the speed of the UAV is of the utmost importance, especially the RSD, in relation to the ship’s SOG. The ship’s data (COG and SOG) are input manually and sent to the UAV from the ground station via an 8Hz data link, so that the drone is aware of the ship’s fixed SOG and COG values. The UAV’s relative speed indicates the rate at which it converges toward the ship’s landing deck. Furthermore, for the effective operation of the landing system, the RSD must be a minimum of 5 knots, ensuring that the clips on the drone’s wings can securely engage with the landing rope.

The lateral position of the fixed-wing UAV with reference to the longitudinal axis of the deck of the ship is an undeniably noteworthy attribute during the landing phase of the drone. The scope of the landing system is to guide the UAV on the lateral midpoint of the helideck, where the SkyHook’s rope is hanging, ready to “catch” the UAV. To achieve this part of the “outer loop” control [15], the BA of the UAV is modified, thereby modifying the angle of turn in the azimuth plane of the drone. In that way, the autonomous landing system guides the UAV along the desired trajectory while rejecting external disturbances, such as wind [15].

The final factor to be considered for a fixed-wing UAV during the execution of a landing on a ship’s deck is the vertical position of the drone, expressed alternatively as its altitude. To reach the desired landing altitude, the UAV must “dive” gradually with a specific rate of descent. This is achieved by controlling the AOD of the fixed-wing drone. Nevertheless, for a successful landing, the UAV must maintain a minimum altitude of approximately 5 m from the landing platform (13 m from the sea surface) to mitigate the risk of the drone colliding with the helideck.

### 3.4. The Landing Process

The small- to medium-sized fixed-wing UAV has completed its mission and is returning to base (RTB). When in range of the TDOA-based UAV localization system, the drone takes its starting position 100 m behind the ship, along the axis of equal distance, where the time difference of arrival and the difference in distance between three of the four nodes in the 2D x-y plane are zero (Figure 2) from 100 ft to 330 ft of altitude. The short distance of 100 m between the ship and the UAV when the landing phase begins is deliberately chosen so that smaller errors occur in the absence of a GPS [15]. At the same time, the data regarding the ship’s constant SOG and COG are transferred via a telecommunication link to the UAV, and the drone adjusts its own speed and course, accordingly, utilizing the INS component.

As the drone follows the landing path with the same course and speed as the ship, the landing phase begins as the fuzzy logic-based autonomous ship deck landing system is enabled. During the approach of the UAV to the landing deck, the FLS controls the three main attributes of landing that are mentioned above to ensure a successful landing, against external factors such as the wind:

1. The RSD must always be greater than the velocity of the ship, so that it is able to approach the landing deck.
2. The lateral movement of the UAV needs to be mitigated by controlling the drone’s BA to ensure that the UAV will follow the landing path.
3. The altitude of the UAV must decrease progressively by controlling the drone’s angle of descent. This is essential to attain a specified height ranging between 5 and 15 m above the landing deck (13 to 23 m above the surface of the sea) facilitating the UAV’s engagement with the “SkyHook” retrieval system’s rope.

Around 20 m from the capture rope, the MilliSign RFID guidance system is enabled and utilized to increase the precision of the autonomous landing system. The UAV then aligns with the batteryless, chipless CR tag and navigates with pinpoint accuracy on the “SkyHook” system. Subsequently, at a relative speed exceeding 5 knots concerning the ship, the UAV maneuvers towards the landing rope and hooks on it.

### 3.5. Electronic Warfare-Resistant Landing

Considerable attention has been paid to the EW resistance of the fuzzy logic-based autonomous ship deck landing system, particularly in military applications, to guide the UAV in a safe and successful landing in a harsh and dense electromagnetic environment. The GPS stands as an important component within the equipment on a UAV, serving as a sophisticated localization mechanism. Its primary function is to provide the drone with vital data regarding its precise position, speed, and height level. However, this pivotal system is susceptible to spoofing and jamming [15]. Lessons learned from modern conflicts around the globe prove that EW, and more specifically, GPS jamming, affects all kinds of civil GPSs, and consequently, counters drone attacks [29]. Considering that civil GPSs are utilized much more frequently than military GPS units in UAVs due to their cost, it is easily understandable that the vulnerability of autonomous UAV systems is real. In modern warfare, EW tactics, such as jamming and spoofing, are utilized by various militaries to neutralize the threat posed by enemy UAVs [30]. To counter these modern warfare operational tactics, this paper proposes the integration of INS sensors with altimeters, anemometers, TDOA-based localization systems, and more equipment onboard UAVs. This integration aims to enhance the estimation of the vehicle’s state and position, even in a hostile electromagnetic environment, in which a GPS-based localization system would be susceptible in spoofing and jamming. Additionally, the selection of short-range sensors utilizing UHF technology, as well as the limited distances that are being used during the landing process, protect the fuzzy logic-based autonomous ship deck landing system from other RF jamming methods and tactics. Furthermore, the exact location of the landing ship is mostly covered during the UAV’s final approach, as emissions from the ship are limited. The working principle of both TDOA and RFID localization systems are based on the UAV’s sensors transmitting the necessary data. Concurrently, the ship’s passive receivers obtain the transmitted data from the UAV, process the information, and finally send the correction directives to the drone via an 8Hz data link. In conclusion, the deliberate omission of GPS, in combination with the utilization of short-range UHF localization systems safeguard the UAV from various EW measures and, at the same time, cover the ship’s position from the enemy signal intelligence (SIGINT) systems.

## 4. The Designed Fuzzy System for Ship Deck Landing

Fuzzy logic “is intended to model logical reasoning with vague or imprecise statements” [31]. Fuzzy set theory provides a framework for dealing with classes of objects in which the boundaries are not precisely defined. Instead of strict, absolute, binary membership (either true or false) [7], fuzzy sets allow for degrees of membership, acknowledging the gradual transition between the two aspects [10].

To construct the fuzzy logic-based autonomous ship deck landing system, five input variables are imported: RLD, AAW, RWS, IAS, and altitude. The input data are received by the airspeed sensor, anemometer, barometric altimeter, and TDOA-based localization system, respectively. Then, the fuzzy logic-based autonomous landing system determines three output variables: RSD, AOD, and BA of the UAV by controlling the drone’s throttle and ailerons.

**Figure 9:**
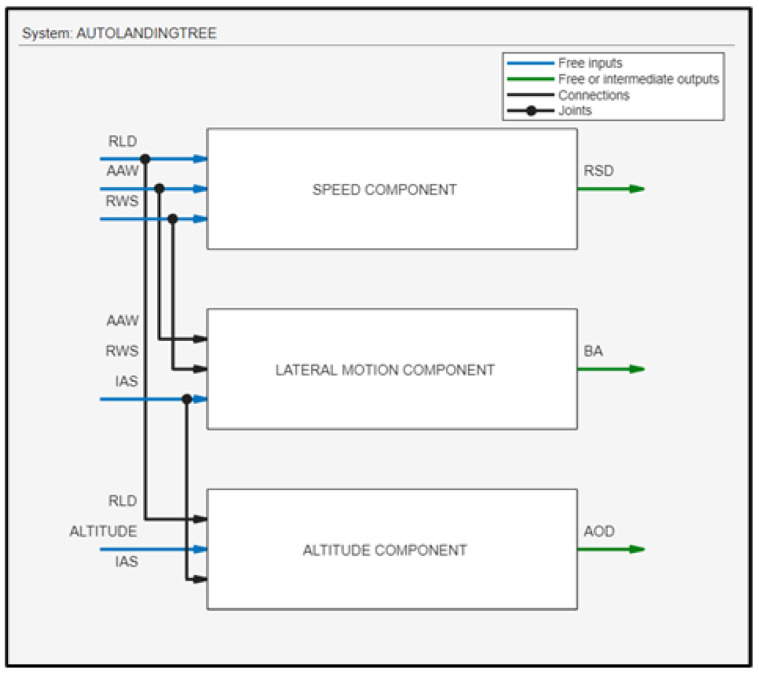
The ship deck landing architecture.

For the FLS, a Mamdani fuzzy inference system was used, which outputs the RSD, BA, and AOD of the fixed-wing UAV. The fuzzy logic-based autonomous ship deck landing system utilizes the input data provided by the range of sensors to calculate the corrective maneuvers of the drone by using three fuzzy logic components. Figure 9 depicts the ship deck landing architecture with an overall presentation of the FLS, where three fuzzy subsystems are interconnected. Each subsystem consists of three inputs and one output; in total, five inputs and three outputs are involved. In the next subsections, an analytical description of these three subsystems is presented.

### 4.1. Speed Component

**Figure 10:**
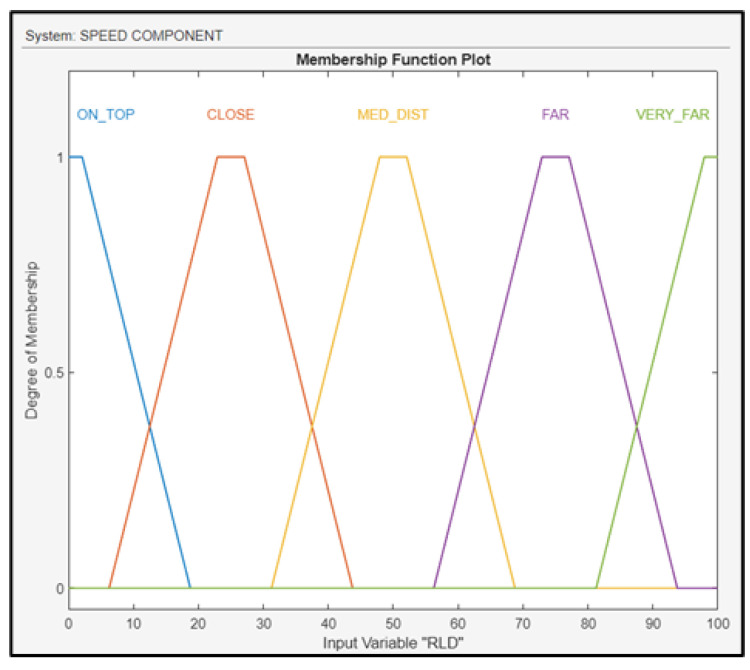
Membership functions for RLD.

The first fuzzy subsystem block is the speed component, with three inputs (RLD, RWS, and AAW) from the landing deck and one output (RSD) with respect to the ship’s velocity. The RLD was fuzzified using linguistic values: on top (ON_TOP), close (CLOSE), medium distance (MED_DIST), far (FAR), and very far (VERY_FAR); the membership functions of RLD are depicted in (Figure 10). The RLD was calculated using the TDOA-based positioning system.

The AAW, in combination with the RWS, immediately affects the RSD. For example, wind from the opposite direction relative to the drone’s motion will result in a degrading effect to the RSD. To facilitate the UAV in continuously reducing the proximity to the ship, it becomes imperative to enhance the drone’s velocity. In addition, establishing a direct correlation between the RSD and the RLD is essential for achieving optimal landing performance.

**Figure 11:**
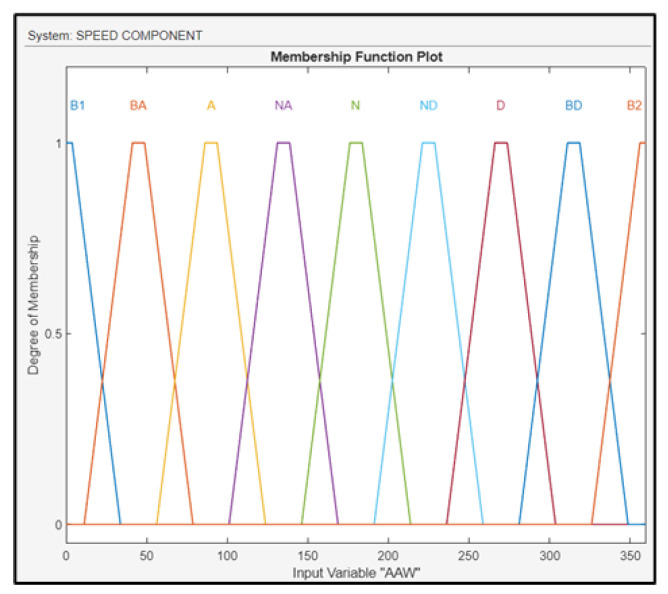
Membership functions for AAW.

The AAW was fuzzified using linguistic values: north (N1 and N2), northeast (NE), east (E), southeast (SE), south (S), southwest (SW), west (W), and northwest (NW), as shown in Figure 11.

**Figure 12:**
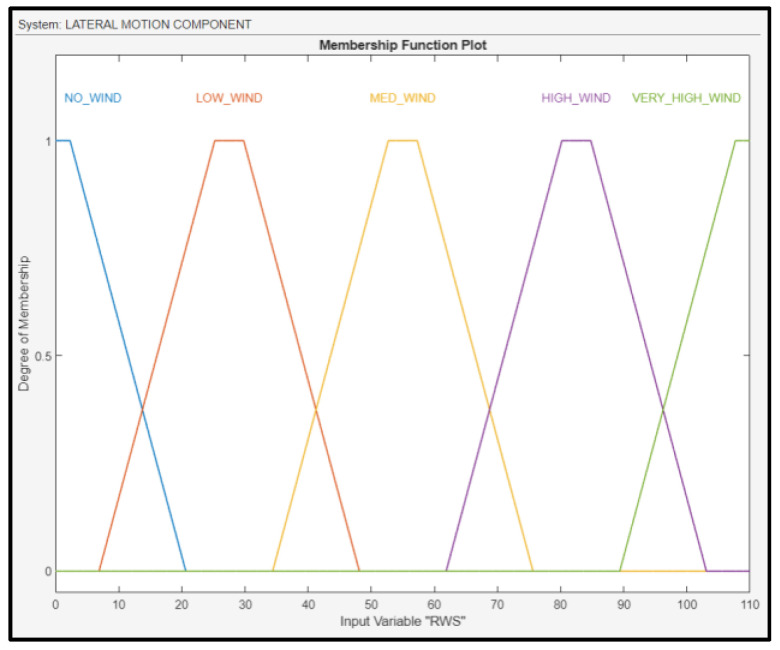
Membership functions for RWS.

RWS was fuzzified using linguistic values: no wind, low wind, medium wind, high wind, and very high wind; their membership functions are shown in Figure 12. WS, as well as the AAW, were calculated using an anemometer.

**Figure 13:**
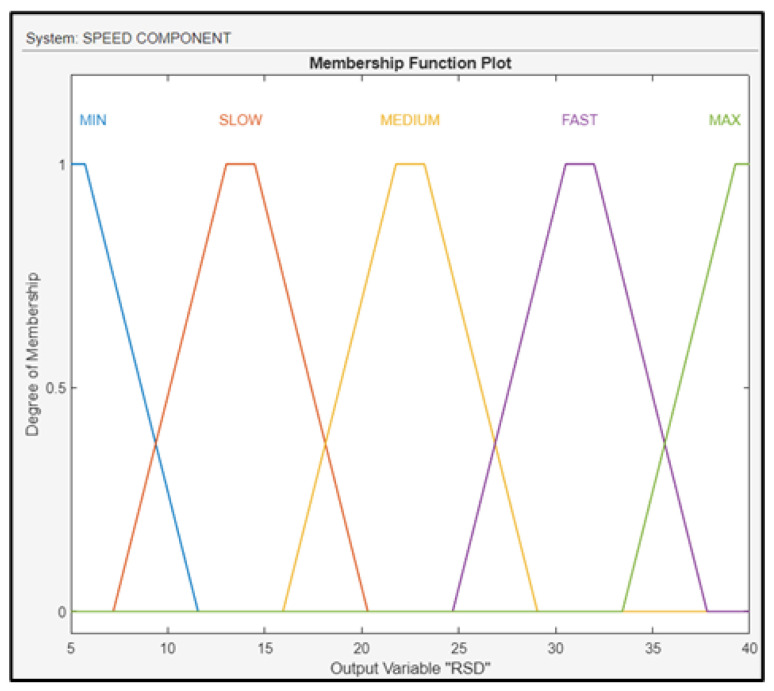
Membership functions for RSD.

The output (RSD) of the fuzzy subsystem was fuzzified using the linguistic values of min, slow, medium, fast, and max, and their membership functions are shown in Figure 13. To control the variable above, the FLS regulates the throttle of the drone.

**Figure 14:**
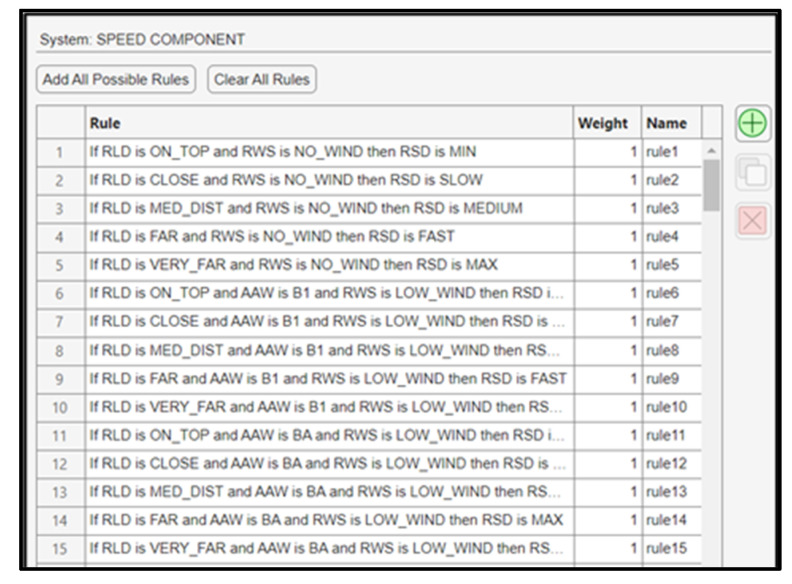
Fuzzy rules for the speed component.

The total number of fuzzy rules is 185, and 15 of them are indicatively presented in Figure 14.

### 4.2. Lateral Motion Component

**Figure 15:**
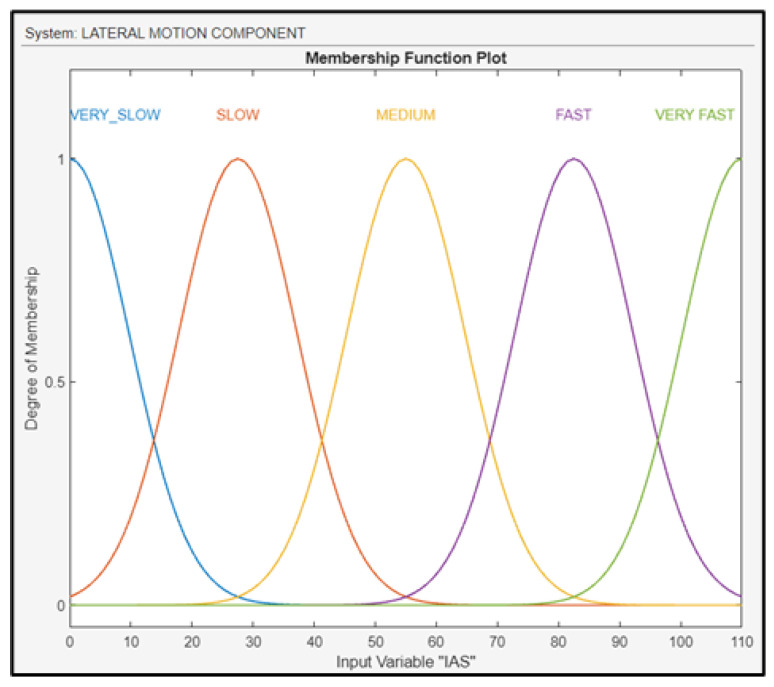
Membership functions for IAS.

The second fuzzy subsystem block is the lateral motion component with three inputs (AAW, RWS, and IA) and one output (BA of the UAV). The interaction of AAW with RWS either facilitates or constrains the UAV’s lateral banking and turning maneuvers. Also, the maneuverability of the drone is strongly dependent on the IAS, which immediately affects the aerodynamics of the UAV. IAS was fuzzified using linguistic values, as shown in Figure 15.

**Figure 16:**
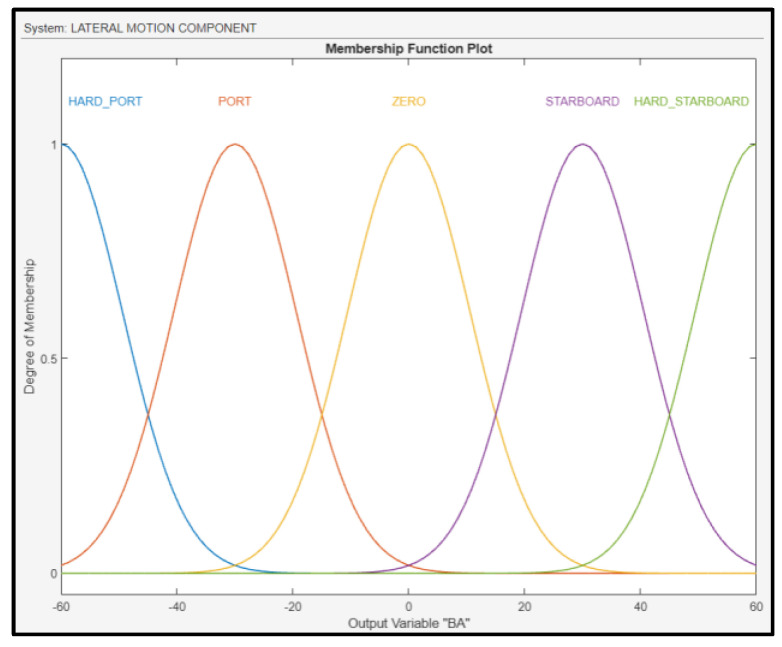
Membership functions for BA.

The output of the fuzzy subsystem is the BA of the UAV, and it was fuzzified as shown in Figure 16. To control the variable above, the FLS regulates the ailerons of the drone’s wings.

**Figure 17:**
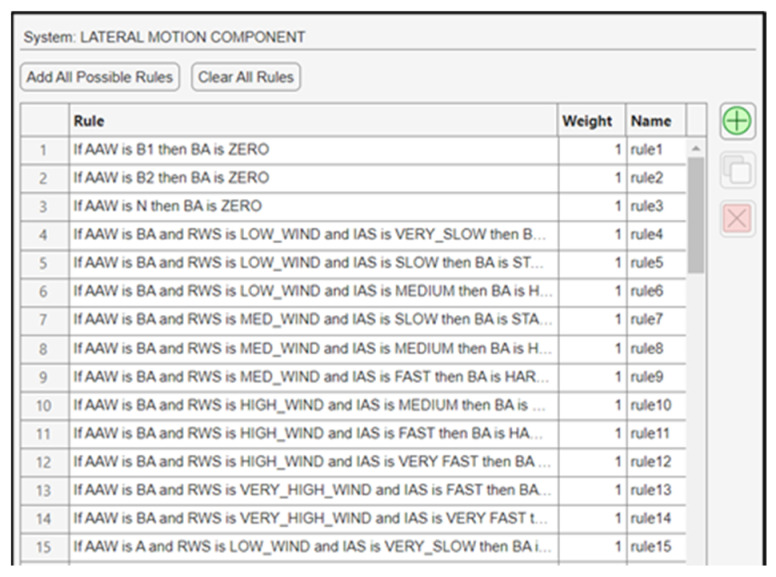
Fuzzy rules for the lateral motion component.

IAS, AAW, and RWS are directly related. When the wind is stronger, then the molecules of air around the body of the vehicle will move faster, depending also on the angle at which these molecules will collide with the UAV’s airspeed sensor. For example, if the wind is blowing against the drone’s course, then the IAS will increase. This occurs because the mass of air that slams the body of the UAV will move faster, leading to better turning maneuverability and lateral banking. For the construction of the fuzzy subsystem, a total number of 82 fuzzy rules were generated (Figure 17).

### 4.3. Altitude Component

**Figure 18:**
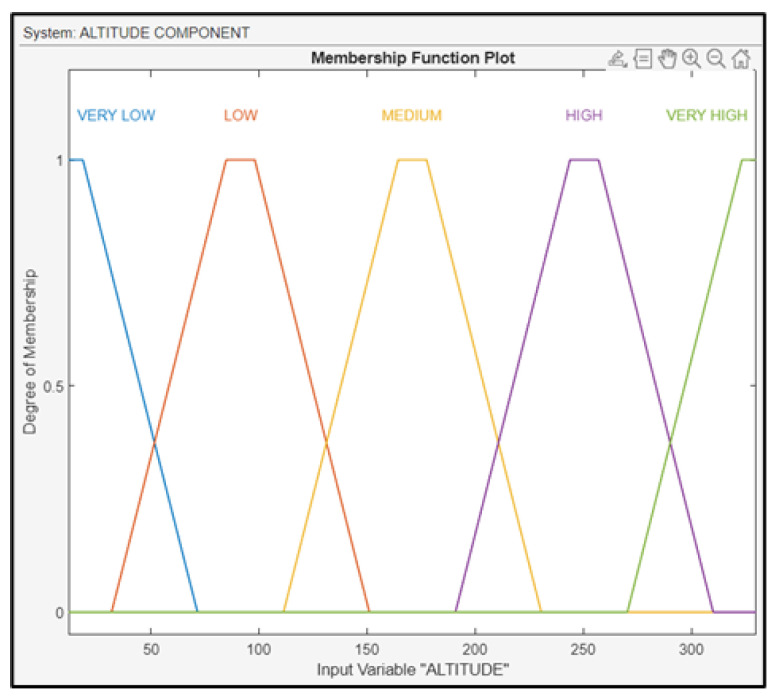
Membership functions for altitude.

The third fuzzy subsystem block is the altitude component, with three inputs (RLD, altitude, and IAS) and one output (AOD of the UAV). As previously mentioned, the maneuverability of the drone, and furthermore the ability of the UAV to make turns in all axes (BA, as well as AOD), is strongly related to the IAS. In addition, achieving a gradual and steady descent of the UAV’s altitude in the final phase of the drone’s approach is crucial for ensuring a successful landing. The altitude was fuzzified using the membership functions depicted in Figure 18.

**Figure 19:**
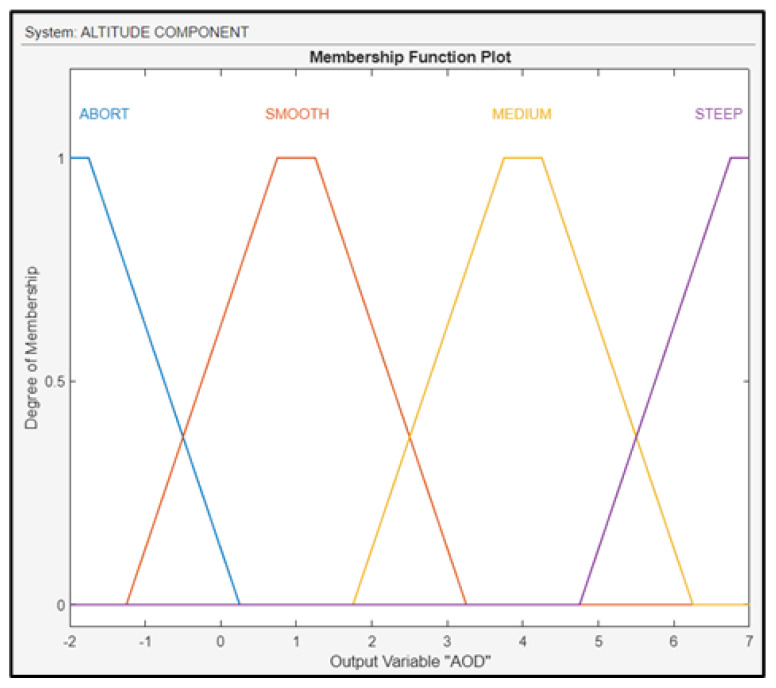
Membership functions for AOD.

The output of the component is the AOD of the UAV, which was fuzzified as shown in Figure 19. To control this variable, the FLS regulates both the ailerons of the drone’s wings, either up or down.

**Figure 20:**
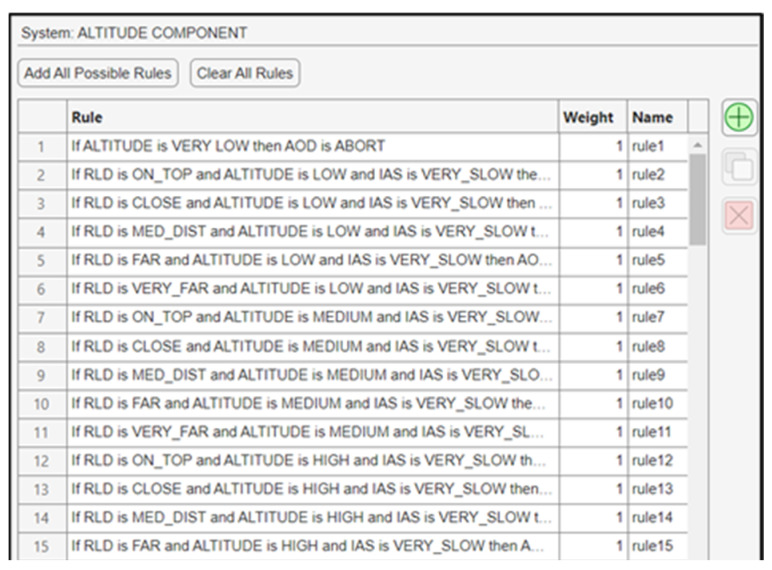
Fuzzy rules for the altitude component.

A total of 105 fuzzy rules were generated, and some of them are shown in Figure 20.

The vertical control and airspeed of a UAV exhibit a reciprocal relationship—when the UAV pitches upward, its speed decreases proportionally, and conversely, pitching downward leads to an increase in speed [4]. Notably, in this study, it is considered that there is no interdependency between airspeed and vertical control.

Additionally, an interdependency between the BA and the AOD was found. For example, if the UAV reduces its height by pitching its nose downwards and at the same time turns right, the combination of these motions would probably result in a different outcome than the desired one. The cause of this paradox lies in the fact that both outcomes of the lateral motion and altitude components of the FLS (BA and AOD) regulate the ailerons of the fixed-wing drone. As a result, conflicting signals from the fuzzy microcontroller may be transmitted to the ailerons, leading to unintended movements of the UAV.

## 5. Simulation Results for Autonomous Landing

To verify the effectiveness of the proposed fuzzy system, a test case is presented separately for each component in the following subsections. This test case involves certain values for the input variables and provides certain values for the three outputs of the fuzzy control system.

### 5.1. Speed Component Results

**Figure 21:**
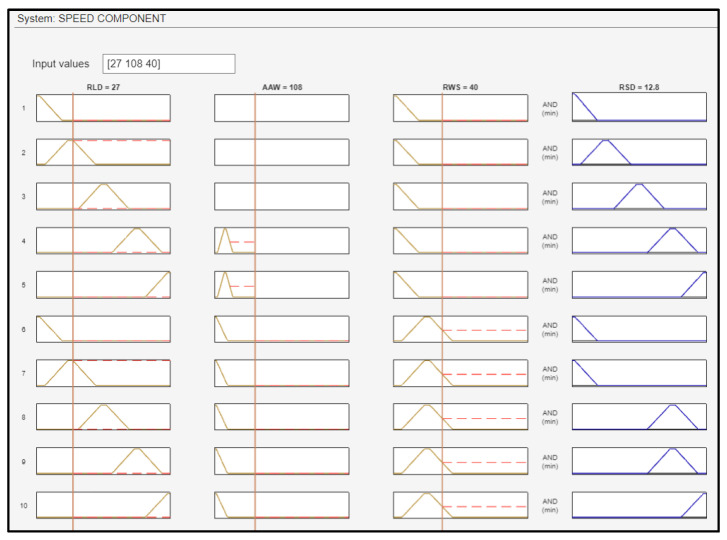
Fuzzy rules activation for the speed component.

**Figure 22:**
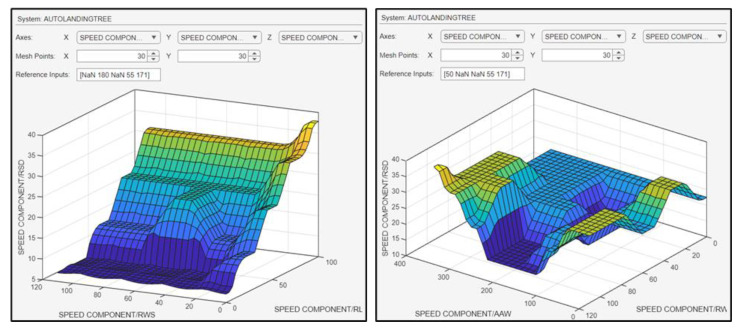
Surface plots indicative of the relationship between the inputs and the RSD.

For the test case of a UAV at a distance of 27 m from the landing deck, with winds blowing from 108 degrees at a speed of 40 knots, the required RSD in relation to the ship’s velocity will be 12.8 knots in order to land effectively onboard the ship. Figure 21 presents the fuzzy rules that were fired using these input data values. Figure 22 shows the surface plots between RSD and the inputs.

The surface plots in Figure 22 indicate the relationship between the RSD and the RWS in combination with the RLD, as well as the relationship between the RSD and the RWS in combination with AAW.

### 5.2. Lateral Motion Component Results

**Figure 23:**
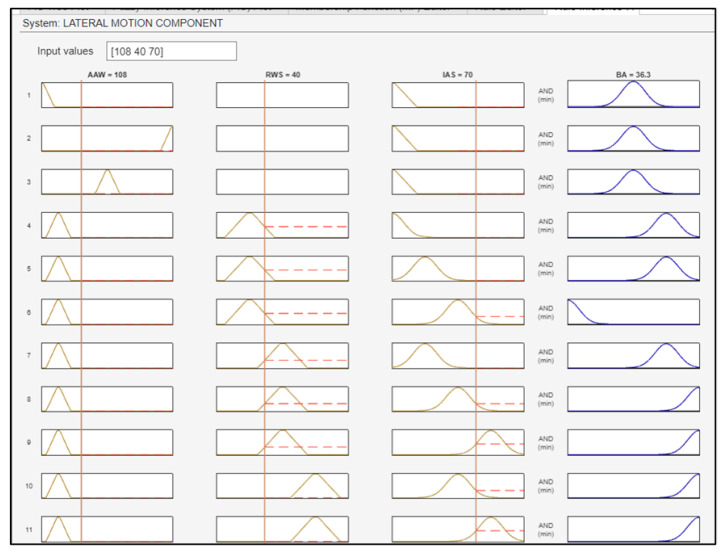
Fuzzy rules activation for the lateral motion component.

**Figure 24:**
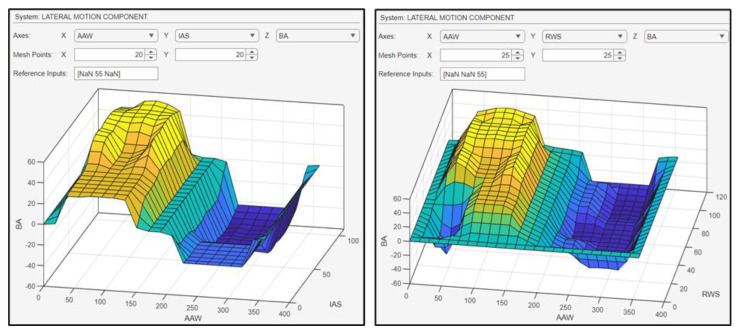
Surface plots indicative of the relationship between the inputs and the BA.

Figure 23 illustrates the case of a UAV flying with an IAS of 70 knots, with winds blowing from 108 degrees at an RSD of 40 knots. As a result, during the turn that the UAV will make to correct its course, a BA of 36.3 degrees will be executed. Figure 24 shows the surface plots between the BA and the inputs.

In Figure 24, on the left surface plot, the correlation between the BA and the inputs AAW and the IAS is presented. The yellow area depicts the starboard turn (BA from 0 to 60 degrees), and the blue area depicts the port turn (BA from 0 to −60 degrees) in relation to the different values of AAW and IAS imported in the FLS. It is evident that a direct correlation exists between IAS and BA: as IAS increases, the drone’s maneuverability also increases, resulting in sharper/steeper turns. On the right surface plot, the interdependency between the BA and the inputs AAW and RWS is shown. Again, the yellow area represents the starboard turn, and the blue area represents the port turn in relation to the inserted values of AAW and RWS. Furthermore, as shown in the surface plot, a direct correlation exists between the RWS and the BA, meaning that as the RWS increases, the UAV is able to take steeper turns.

### 5.3. Altitude Component

**Figure 25:**
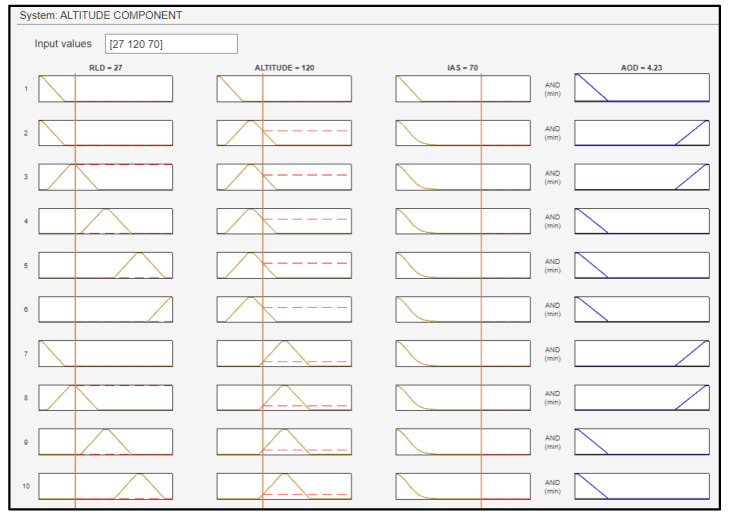
Fuzzy rules activation for the altitude component.

**Figure 26:**
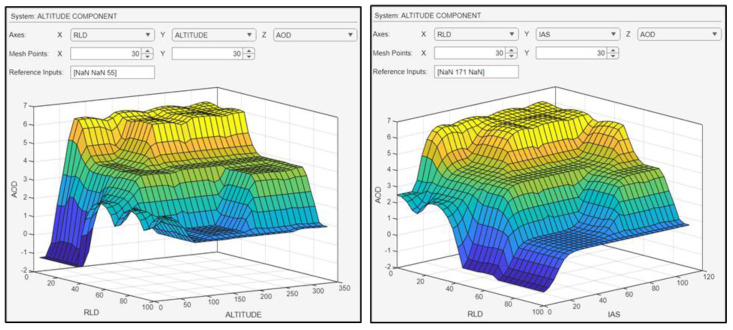
Surface plots indicative of the relationship between the inputs and the AOD.

The UAV flies with an IAS of 70 knots at a range of 27 m from the landing deck at a height level (altitude) of 120 feet. Consequently, the yielded AOD of the UAV was equal to 4.23 degrees (Figure 25), ensuring a sufficient altitude for a successful landing on the “SkyHook” capture system. Figure 26 shows the surface plots between the AOD and the inputs.

Figure 26 illustrates the surface of the angle of descent. On the x axis, the range from the landing deck is shown (in meters), and on the y axis is the airspeed (in knots).

The surface plots of Figure 26 indicate the relationship between the AOD and its inputs. On the left plot, the correlation between the AOD and the inputs of altitude and RLD is shown. The further the drone is from the ship, the greater the AOD, and the higher the altitude, the steeper the dive. On the right plot, the interdependency of the AOD and the inputs RLD and IAS is presented. As the IAS increases, the ability of the UAV to maneuver is greater, and as the distance of the drone from the ship decreases, the smaller the AOD becomes.

In conclusion, in the case of a UAV operating at a distance of 27 m from the landing deck at an altitude of 120 feet with winds blowing from 108 degrees at a speed of 40 knots and an IAS of 70 knots, the required RSD in relation to the ship’s velocity is 12.8 knots. During the turn that will be executed, the UAV will have a BA of 36.3 degrees, and the AOD will have a value of 4.23 degrees.

### 5.4. Data Analysis

**Figure 27:**
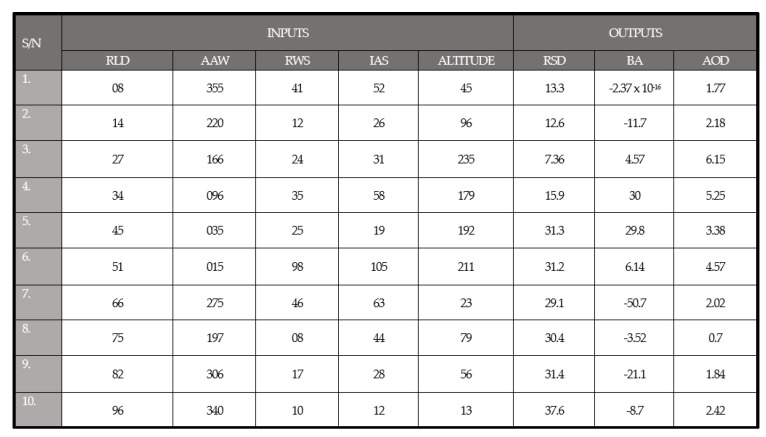
Input–output data of the FLS.

To test the reliability and effectiveness of the FLS, a certain number of different input values [18] was imported in the rule inference tab of the FuzzyLogicDesigner Toolbox in MATLAB software. The output results are shown in Figure 27.

By observing the results in Figure 27, certain conclusions can be drawn. At first, the further the UAV is from the ship, the greater the RSD in order to cover the distance to the landing platform as fast as possible. Additionally, when the direction of the wind is opposite to the direction of the drone, the RSD increases, which is in contrast to when the vectors of the wind and the drone are parallel and the RSD decreases. Regarding the BA, the maximum values are found when the AAW is 090 or 270; in other words, when the wind is eastern or western in relation to the UAV’s course, the minimum values are found when the AAW is 0 or 180, northern or southern to the drone’s direction. The BA, however, is immediately affected by the RWS; the greater the wind speed, the harder the turn of the UAV. The AOD, on the other hand, maximizes as the altitude and RLD increase. Lastly, the IAS is utilized as a measure of the aerodynamic maneuverability and stability of the UAV. The greater the value of IAS, the greater the ability of the drone to make steeper turns (greater BA) and harder dives (greater AOD).

## 6. Conclusions

The primary aim of this research was to design a fuzzy logic-based autonomous ship deck landing system specifically tailored for medium- and small-class fixed-wing UAVs. This system aims to alleviate maritime UAV operators from the demanding and challenging task of landing a drone on a moving ship. A variety of sensors onboard the UAV and the ship has been utilized, including RF localization systems such as RFID and TDOA-based mechanisms, anemometers, altimeters, and airspeed sensors. However, a key objective of this study was to reduce the requisite number of sensors and equipment, with a dual focus on cost-effectiveness and diminished system complexity. Additionally, particular attention has been given to the deliberate exclusion of GPS, thus enhancing the UAV’s resistance to long-range EW jamming and spoofing techniques.

The purpose of the autonomous ship deck landing system is to control the UAV’s movement in the three-dimensional space, ensuring precise positional control during the final approach. The wind, being an external factor that directly influences drone landing, particularly at sea, was integrated into the fuzzy logic-based system along with the airspeed of the UAV, altitude, and distance from the landing platform. This integration was facilitated through the UAV’s sensors. The FLS, in return, provides as outputs the RSD in relation with the speed of the ship, the angle of descent, and the bank angle of the drone, or alternatively, the azimuth angle of turn, to control the UAV’s movement. As a result, the simulation results are generally satisfactory, affirming the reliability of the autonomous fuzzy logic landing system.

However, this paper does not specifically address the pitch and roll aspects of the vessel in the context of ship deck landings. The dynamic movement of the landing platform in the 3D axis is very difficult to predict for the UAV to land onboard the helideck. Although this limitation is countered by the utilization of the “Skyhook” system, further research on this topic would definitely improve the UAV landing experience. Additionally, certain assumptions were made during the research process, including the correlation between airspeed and vertical control and the interdependency between the BA and the AOD, as mentioned in Section 4.3.

Lastly, suggestions for future research include searching for more optimal membership function parameters and rule base by incorporating the knowledge and the experience of maritime UAV operators and maritime helicopter pilots, who undeniably excel in harsh, all-weather landings onboard ships. This data could be collected using polls or other statistic methods. Research could also explore additional input variables or parameters, such as humidity or visibility (particularly relevant when utilizing computer vision algorithms for landing). These factors significantly impact UAV navigation and control during the final stage of flight, where environmental conditions and the drone’s aerodynamics differ significantly from the usual phases of UAV flight.

## Data Availability

Data are contained within the article.
